# Postmortem Cerebrospinal Fluid Pleocytosis: A Marker of Inflammation or Postmortem Artifact?

**DOI:** 10.1155/2012/964074

**Published:** 2012-02-27

**Authors:** James A. Morris, Linda M. Harrison, David R. Telford

**Affiliations:** ^1^University Hospitals of Morecambe Bay NHS Foundation Trust, Royal Lancaster Infirmary, Ashton Road, Lancaster, LA1 4RP, UK; ^2^Department of Pathology, University Hospitals of Morecambe Bay NHS Foundation Trust, Royal Lancaster Infirmary, Lancaster LA1 4RP, UK

## Abstract

The aim of this paper is to reassess the significance of postmortem cerebrospinal fluid pleocytosis. Published articles of CSF changes after death were reviewed, and reanalysis, in the light of modern views on the significance of bacterial postmortem isolates, was undertaken. There is theoretical and experimental evidence that the blood brain barrier to the movement of protein and cells is preserved in the first few hours after death. The number of mononuclear cells in the cerebrospinal fluid does rise in the first 24 hours after death, and this is most probably due to detachment of leptomeningeal lining cells. But the marked increase in lymphocyte counts seen in some cases of sudden infant death syndrome (SIDS) and in other deaths in the paediatric age range could well be a marker of inflammation.

## 1. Introduction

In a healthy individual, the cerebrospinal fluid (CSF) is clear and colourless. It has a low concentration of protein and contains very few cells. The composition is different than that of the blood due to a highly selective blood brain barrier. Any increase in cellular content or protein composition of the cerebrospinal fluid (CSF) above the normal range for the specific age group is an absolute indicator of meningeal disease (inflammation) [1]. But after death the number of cells in the CSF rises, even in the absence of evidence of meningitis, and this is viewed as a postmortem artifact [2, 3]. Thus counting cells in CSF obtained postmortem is not considered to be useful in diagnosis. In this paper we examine the evidence for this assumption.

## 2. Studies of Postmortem CSF Pleocytosis

Platt and colleagues obtained postmortem specimens of CSF from 26 cases of sudden infant death syndrome (SIDS), 24 paediatric deaths from a hospital setting, and 14 adult deaths [2]. The age range, the postmortem interval, and the CSF cell count per cubic mm are shown in Table 1.

The CSF cell count in the SIDS cases varied from 37 to 3250 cells per cubic mm (mean 647). These counts were significantly higher (*P* < 0.005) than in the hospital paediatric group and the adult group. The cells were mononuclear; the authors do not record the presence of neutrophil polymorphs in any of the cases. The typeable cells were 60–70% macrophages and 20–40% lymphocytes, but beyond 12 hours postmortem, the cells became vacuolated and were difficult to type. It is stated that “none of the postmortem cultures grew bacteria, and sections of the brain revealed no inflammation.”

Wyler and colleagues obtained CSF by lumbar puncture from 69 adult deaths aged 16 to 90 years [3]. Thirty five of the corpses were stored at room temperature (20°C) and 34 were placed in cold storage (4°C) shortly after death. The specimens of CSF were obtained between 3 and 39 hours for the 35 cases stored at room temperature and 3 and 53 hours for those in cold storage. The CSF cell count increased with postmortem interval and the rate of increase was more marked for the bodies stored at room temperature. Part of the data is presented in Table 2.

In this study, the CSF cell count in the first few hours after death was only slightly raised above the levels regarded as normal in life, and even after 24 hours was below 100 cells per cubic mm. These results are similar to those obtained by Platt et al. [2] in adults, but the CSF cell counts are significantly less than those seen in SIDS and in the paediatric non-SIDS deaths.

## 3. The Blood Brain Barrier

The anatomy and physiology of the blood brain barrier is reviewed in detail by Fishman [1] in his comprehensive treatise and more recently by Ballabh et al. [4] and Abbott et al. [5]. The leptomeninges have an outer layer of arachnoid mater and an inner layer of pia mater. These membranes are formed of connective tissue, and the subarachnoid space that lies between these layers is traversed by connective tissue trabecula. There are cells lining the pia and arachnoid membranes and desquamation of these cells postmortem could have contributed to the CSF mononuclear cells observed in the above studies by Platt et al. [2] and Wyler et al., [3]. 

 The main barrier to the transfer of protein and cells between the blood and CSF is the endothelium of the cerebral capillaries [4, 5]. The brain capillaries are different than most systemic capillary vessels in that the endothelial cells have tight junctions and transfer of material is through the cells rather than between the cells. Furthermore, the brain endothelial cells have more mitochondria than systemic vessels, and this reflects the additional energy required to pump protein and other chemicals from the lumen through the cell cytoplasm rather than relying on passive diffusion between leaky cell junctions or through endothelial fenestrations. The endothelial cells, however, have lower energy requirements than brain neurons and the brain cells die first if the oxygen supply is disrupted. 

Lymphocytes pass through the endothelial cells by emperipolesis; this is a complex process in which the inflammatory cell invaginates into the endothelial cell and literally passes through to emerge intact at the opposite side [1, 5–7]. The process depends on the active cooperation of both cells, and it is difficult to imagine how it could occur after death. Inflammation in the systemic circulation leads to increased permeability of the vasculature and protein molecules and cells pass between the endothelial cells. If inflammation involves the meninges the tight junctions might open to allow the egress of neutrophils, mononuclear cells, and proteins, but this does not occur in the absence of inflammation [4, 5]. The pro-inflammatory cytokines TNF-alpha and IL1-beta increase permeability of the blood brain barrier and disrupt tight junctions [4]. These cytokines are released in infection and as a consequence of hypoxic ischaemic change. 

There are capillary vessels within the dura mater, which are similar to systemic capillaries in having leaky junctions and fenestrations. Protein and cells could cross these vessels passively but the arachnoid lining cells have tight junctions and maintain a barrier to diffusion into the CSF [4, 5]. 

In the ventricles, the choroids plexus is the site of transfer of protein and other chemicals into the CSF. The capillaries in the choroids plexus resemble endothelial cells elsewhere in the body but there are tight junctions between the lining ependymal cells of the choroids plexus, which maintain the blood brain barrier at this level. [1, 4, 5]. 

An interruption to the oxygen supply to the brain will cause cessation of neuronal function and death will ensue. The activity of the cerebral endothelial cells will decrease and the transfer of protein and other substances will be brought to a halt. However, the endothelial cells will survive intact for some time and at least initially we would not expect to see any leakage of protein or cells into the CSF from the cerebral circulation.

## 4. Changes in CSF Protein after Death

The normal range of CSF protein is shown in Table 3. However, the protein concentration varies not only with age but also to a small extent by site. It is lowest in the ventricular CSF, intermediate in the cisterna magna, and highest in the lumbar region. The difference between the cisterna magna and lumber region is of order 0.1 g/L. 

The blood brain barrier to the free movement of protein was first defined by Ehrlich [8] and his students. They injected aniline dyes intravenously in experimental animals and noted that at postmortem the tissues were stained blue but the CSF and the brain were unstained [1]. The aniline dyes attached to albumin and, therefore, the stain reflected the distribution of albumin in the body. The fact that the differential staining was noted at autopsy indicates that the blood brain barrier was maintained after death for at least a short interval. 

Mangin and colleagues investigated CSF protein levels in 44 cadavers aged 5 to 74 years. CSF was obtained by cisternal puncture between 3 and 24 hours after death [9]. They divided their cases into three groups.

**Group**

15 subjects who died suddenly (less than 10 minutes) due to homicidal firearm wounds, stabwounds, or hanging. The mean CSF protein level in these cases was 0.373 g/L with a standard deviation of 0.181 g/L.

**Group**

13 subjects whose “death agony duration lasted between 10 minutes and 6 hours.” No more clinical information is given but these are cases who became acutely unwell and died within 6 hours of the onset of the illness. The CSF protein in this group was 1.546 ± 0.46 g/L (mean ± SD).

**Group**

16 subjects whose “death agony duration was more than 6 hours” and who died in spite of intensive care. The CSF protein was 8.267 ± 6.249 g/L.

These results indicate that if death is rapid and the CSF protein is normal at the time of death, then it will remain within the normal range for a few hours after death. In those first few hours there is no significant passive leakage because the endothelial cells and their tight junctions are still intact. However, over a period of 24 hours the endothelial cells will start to lyse, the junctions will start to leak, and passive transfer will gradually ensue. The passive leakage involves proteins to a small extent but not red cells and by inference not white cells. In groups 2 and 3, the subjects were unwell prior to death and the most likely explanation is production of cytokines as part of the illness causing a generalized inflammatory process throughout the body including the blood brain barrier [4]. The movement of proteins, and cells, would be an active process in the brain requiring intact and functioning brain capillary endothelial cells but with some loss of permeability due to cytokine secretion. Following death, however, the endothelial cells might well lyse more quickly and passive leakage of protein would ensue within 24 hours. Thus the raised levels of CSF protein noted in groups 2 and 3 are probably a combination of increased levels prior to death and further increases after death. The authors did not count cells in the CSF samples, but the inference we can draw is that there would be few or no lymphocytes in group 1 but there could well have been lymphocytes and even neutrophils present in groups 2 and 3 as the proinflammatory cytokines TNF-alpha and IL1-beta increase leukocyte transmigration as well as increase blood brain barrier permeability and disrupt tight junctions [4]. 

Osuna and colleagues also studied CSF protein levels in autopsy cases [10]. They examined 11 cases of head trauma, 7 cases of hypoxia (4 carbon monoxide or drug poisonings and 3 hangings), 7 sudden cardiac deaths, and 9 others (natural and unnatural). The CSF samples were obtained by cisternal puncture. A number of biochemical investigations were done including CSF protein. The results are shown in Table 4. The CSF levels are markedly raised in all cases and are of no value in diagnosis. But the key difference between this paper and that of Mangin et al. [9] is the postmortem interval. It would appear that the CSF protein leak starts in the first 24 hours but gathers speed thereafter. 

## 5. Correlation of CSF Cell Counts with Histological Assessment of the Meninges

In the study by Platt et al. [2], there was no histological evidence of meningitis even in cases with over 3000 mononuclear cells per cubic mm of CSF. Since the CSF bathes the leptomeninges, we would expect to see a similar concentration of cells in the meninges at autopsy as were found in the CSF [11]. But what exactly is the correspondence between the CSF cell count determined objectively and our subjective impressions of the mononuclear content of the meninges assessed by histology? The mean diameter of a high power field is 0.5 mm and the thickness of a histological section is 0.005 mm. Thus if 800 cells of the size of a lymphocyte or a neutrophil polymorph were distributed in one cubic mm of tissue and the tissue were serially sectioned, we would have 200 histological sections each with 4 high power fields. There would be one inflammatory cell per high power field (This assumes we count all the cells and never count a cell twice. In fact, we would miss some cells and count some twice but the former error is likely to exceed the latter and therefore the one per high power field estimate will be, if anything, too high). Thus even 3000 mononuclear cells per cubic mm of CSF will mean fewer than 4 cells per high power field; not a number that would lead to a diagnosis of meningitis [11]. But 10 lymphocytes per cubic mm of CSF in life would be enough for a diagnosis of lymphocytic meningitis in its earliest stages. Thus an objective count of mononuclear cells in the CSF will be a far more sensitive way of diagnosing meningitis than histological examination of the meninges.

## 6. Infection and SIDS

In the publication by Platt et al. [2], the following statement occurs in the discussion “It is clear from this study that children and adults develop a postmortem CSF pleocytosis. However, the degree of pleocytosis is significantly greater in children, particularly in cases of SIDS.” The assumption that the CSF pleocytosis is a postmortem artifact is based on two factors. The first is that there was no evidence of meningitis as assessed histologically. The second is that there was no evidence of any other infection at autopsy to explain the sudden infant death. Neither of these assumptions is any longer valid. Counting cells in the CSF is a more sensitive way of diagnosing meningitis than is examination of histological sections of meninges. And there is now a considerable body of evidence that some cases of SIDS are caused by bacterial infection even if the autopsy is completely negative. 

The latest in a long line of publications linking bacterial infection and SIDS appeared in the Lancet in 2008 [12]. A group from Great Ormond Street undertook a retrospective review of 546 cases of sudden unexpected death in infancy (SUDI) examined at their institution over 10 years. They found that isolation of *Staphylococcus aureus *and *Escherichia coli *from lung, blood, spleen, or CSF was more common in unexplained SUDI (synonymous with the term SIDS as used in the Platt et al. study, [2]) than in cases of SUDI explained by a noninfective cause. *S. aureus *was found in 16% of cultures from unexplained SUDI compared with 9% of cultures from explained, noninfective, SUDI (*P* = 0.005). *E. coli *was found in 6% of cultures from unexplained SUDI compared with 1% of cultures from explained noninfective SUDI (*P* = 0.003). The authors are cautious in their analysis and emphasise that they have shown an association and not proven a causal link. But other evidence from a number of studies, reviewed in a lead article in the same issue of the Lancet, supports this link [13]. The organisms *S. aureus *and *E. coli *have been linked to unexplained SUDI in a number of studies extending back to 1987 [14]. There is now sufficient evidence to regard these organisms as a possible cause of some cases of unexplained SUDI [15–17]. 

The mode of death in unexplained SUDI is not known but we do know that it is rapid in many cases. In the CESDI SUDI study carried out in England between 1993 and 1996, a total of 318 cases of unexplained SUDI were investigated [18]. The majority (76.8%) appeared to be well when last seen, that is, they had a Cambridge Baby Check score of between 0 and 7 in the 24 hours prior to death. Amongst the daytime deaths in this group, 38% were observed alive 30 minutes prior to discovery and 9% within 10 minutes of discovery [19]. In the small number of infants who have died whilst on a monitor, the physiological changes of progressive hypoxia, bradycardia, and finally cardiorespiratory arrest take less than 20 minutes [20]. If bacteria such as *S. aureus *and *E. coli *present in the blood, but in the absence of overt inflammation, can cause death in a short interval, the possible mechanisms include the following.

- The release of toxins, which directly combine with neural and or cardiac membranes and interfere with homeostatic control of cardiorespiratory function.
- A heightened or abnormal host response to the bacteria with lymphocyte proliferation, cytokine release, and generalized but subtle signs of inflammation. But since lymphocyte proliferation takes time, there would have to be a subclinical prodromal period prior to the dramatic final episode.

A specific mechanism that includes both the above possibilities is the release of pyrogenic toxins (such as toxic shock syndrome toxin [TSST] and staphylococcal enterotoxin C [SEC]) by *S. aureus *leading to the explosive release of cytokines from lymphocytes primed by previous episodes of staphylococcal bacteraemia. The cytokine cascade causes toxic shock and a transfer of protein and cells across capillary cell walls in both the systemic and cerebral circulations.

## 7. Discussion

The morphology, physiology, and pathology of the blood-brain barrier has been studied in detail in animal models and in human subjects. Fishman published a comprehensive review in 1992 and since then there have been further reviews by Ballabh et al. [4] and Abbott et al. [5]. However, there is a paucity of information on changes in the blood-brain barrier after death in human subjects, and pathologists have been too ready to dismiss the changes noted in the CSF as a postmortem artefact. 

In life, a normal specimen of CSF contains no more than 2 to 4 mononuclear cells per cubic mm and no neutrophil polymorphs. If more cells are found, the sample is not normal and CSF pleocytosis is diagnostic of meningeal inflammation as long as an acute CSF bleed can be excluded [1]. However, the studies of Platt et al. [2] and Wyler et al. [3] show that a CSF pleocytosis does occur as a postmortem artifact. In adults, the number of mononuclear cells in the CSF gradually increases over the first 24 hours. This plainly is a consequence of postmortem events and does not indicate or reflect the number of cells in the CSF prior to death. The most likely explanation is that cells lining the leptomeninges progressively detach with time after death and float into the CSF. But it is much more difficult to postulate a mechanism by which lymphocytes can enter the CSF from the blood after death. 

If death is sudden, the endothelial lining cells of the cerebral blood vessels will remain intact for a few hours at least. This is what we would expect from our knowledge of their anatomy and physiology. It is also what was observed by Mangin et al. [9] in their study of CSF protein in cases of sudden death due to shooting, stabbing, and hanging. In those cases, there was not a significant rise in CSF protein in the first 24 hours after death. Lymphocytes pass from the cerebral blood to the CSF by a process of emperipolesis [1, 5–7]. This is a complex movement through the cell in which the endothelial cytoplasm wraps around the lymphocyte. There is no evidence of which we are aware that this process can occur after death. It is theoretically possible that lymphocytes could exit from blood vessels in the dura mater and track through the arachnoid mater into the CSF by a passive process. Or indeed pass from capillaries in the choroids plexus through into the ventricles. But we would expect them to be prevented by tight junctions in arachnoid lining cells and in choroid plexus ependymal cells in the first few hours after death [5, 11]. Indeed the observation that the CSF protein does not rise in the first few hours after death argues against this route. The CSF protein does rise eventually in all cases as shown by the study of Osuna et al. [10], but it is a protein that leaks not red cells. If red cells do not move passively into the CSF even after 48 hours, then one would not expect the larger white cells to move in this way. Furthermore, Platt et al. [2] did not record the presence of neutrophil polymorphs in the CSF, which would be unusual if the lymphocyte entry was purely passive. 

In summary, if the lymphocyte entry is passive one would expect to see red cells and neutrophil polymorphs as well as mononuclear cells and a much more marked increase in CSF protein than was observed in the Mangin et al. [9] study. It is difficult to see how the process could be active after death as endothelial cells depend on energy from mitochondria to move cells and protein into the CSF. That should cease at the time of death. The final possibility is that the lymphocytes seen in SIDS cases and in the paediatric hospital death group in the Platt et al. [2] study were in fact present at the time of death and indicate inflammation. 

There were two reasons for regarding the lymphocytes seen in the SIDS cases as a postmortem artifact. The first was that there was no evidence of meningitis as assessed by histology. The second was that there was no evidence of infection as the cause of death. The first objection is no longer valid because simple calculations indicate that counting cells in the CSF is a much more sensitive indicator of meningitis than is histological assessment of the meninges. The second objection is also not valid because there is now a considerable body of evidence pointing to an infectious aetiology of SIDS [12–17]. 

It is time to reassess the pathogenic significance of lymphocytes seen in postmortem CSF samples. We need to collect CSF samples in the first few hours after death and examine them urgently as we would in life. A cell count is essential but we should also use the full armory of modern immunohistological techniques with a wide range of cluster differentiation (CD) markers to differentiate lymphocytes from other mononuclear cells. We need to compare unexplained SUDI with cases in which an explained noninfective cause appears likely. We should also investigate deaths at other ages, not just the paediatric age range but also adults. Bacteria are commonly found at autopsy and too readily dismissed as contaminants or as the consequence of agonal events [21]. It could well be that episodes of bacteraemia are a common last event on the road to death in a range of conditions. 

Studies are planned, and in some cases underway, to investigate unexplained SUDI using the full array of modern molecular techniques. These include microarray analysis to identify genetic signatures of the host response to infection as well as genetic signatures to mark the presence of pathogens. Proteomic analysis of fluids for bacterial toxins is another area of considerable promise. But we should not forget something which is much simpler-counting lymphocytes in the CSF.

## Figures and Tables

**Table 1:** Data from Platt et al [2].

CASES	Number of cases	Age range	Postmortem interval (mean)	CSF cell count per cubic mm, range (mean)
SIDS	26	5 weeks–5 months	2–28 hours (16.1)	37–3250 (647)
Hospital paediatric gp.	24	1 day–16 years	1.5–22 hours (10.58)	0–593 (141)
Adults	14	30–74 years	5–48 hours (15.14)	1–108 (28)

**Table 2:** Data from Wyler et al [3].

Postmortem interval	Number of cases	CSF cell count per cubic mm	CSF cell count-mean
0–6 hours	12	3–19	7.8
0–5 hours	11	3–45	17.9
12–24 hours	29	5–81	31.6

**Table 3:** Data adapted from Fishman [1].

Age	CSF protein g/L
Neonate	0.2–1.7
1–30 days	0.2–1.5
30–90 days	0.2–1.0
3–6 months	0.15–0.5
6 months–10 years	0.15–0.3
Adult	0.2–0.45

**Table 4:** Data from Osuna et al [10].

Diagnostic group	Number of cases	Survival time in hours, mean (SD)	Postmortem interval in hours, mean (SD)	CSF protein in g/L, range (median)
Head trauma	11	0.9 (0.8)	51.5 (18.8)	2.8–42.4 (14)
Hypoxia	7	1.0 (1.0)	49.7 (23.3)	2.4–28.5 (7)
Sudden cardiac death	7	0.0 (0.0)	41.1 (26.9)	0.5–6.1 (2)
Other	9	2.0 (1.7)	51.2 (30.5)	1.2–9.2 (6)
